# Identification of macrophage-related genes in sepsis-induced ARDS using bioinformatics and machine learning

**DOI:** 10.1038/s41598-023-37162-5

**Published:** 2023-06-19

**Authors:** Qiuyue Li, Hongyu Zheng, Bing Chen

**Affiliations:** 1 Department of Emergency Medicine, The Second Hospital of Tianjin Medical University, No. 23, Pingjiang Road, Hexi District, Tianjin, 300211 China; 2 Department of Maxillofacial Surgery, The First Affiliated Hospital of Chongqing Medical University, No. 1, Youyi Road, Yuzhong District, Chongqing, 400016 China

**Keywords:** Computational biology and bioinformatics, Data integration, Data mining, Data processing, Databases, Functional clustering, Gene ontology, Genome informatics, Machine learning, Bacterial infection, Respiratory distress syndrome, Diagnostic markers, Predictive markers, Infection

## Abstract

Sepsis-induced acute respiratory distress syndrome (ARDS) is one of the leading causes of death in critically ill patients, and macrophages play very important roles in the pathogenesis and treatment of sepsis-induced ARDS. The aim of this study was to screen macrophage-related biomarkers for the diagnosis and treatment of sepsis-induced ARDS by bioinformatics and machine learning algorithms. A dataset including gene expression profiles of sepsis-induced ARDS patients and healthy controls was downloaded from the gene expression omnibus database. The limma package was used to screen 325 differentially expressed genes, and enrichment analysis suggested enrichment mainly in immune-related pathways and reactive oxygen metabolism pathways. The level of immune cell infiltration was analysed using the ssGSEA method, and then 506 macrophage-related genes were screened using WGCNA; 48 showed differential expression. PPI analysis was also performed. SVM-RFE and random forest map analysis were used to screen 10 genes. Three key genes, SGK1, DYSF and MSRB1, were obtained after validation with external datasets. ROC curves suggested that all three genes had good diagnostic efficacy. The nomogram model consisting of the three genes also had good diagnostic efficacy. This study provides new targets for the early diagnosis of sepsis-induced ARDS.

## Introduction

Sepsis is defined as life-threatening organ dysfunction caused by a dysregulated host response to infection^1^. Despite significant advances in treatment, sepsis affects approximately 19–48.9 million people worldwide each year and remains one of the leading causes of death in critically ill patients worldwide^2^. Sepsis causes various complications, such as lung injury, liver injury, kidney injury, myocardial injury and brain injury^3–5^. Acute respiratory distress syndrome (ARDS) is a common complication in patients with sepsis and is characterized by diffuse alveolar injury; patients present with clinical symptoms such as acute respiratory distress, hypoxemia and pulmonary oedema^6^. The mortality rate of sepsis-induced ARDS is 30–40%, which is higher than that of other types of ARDS^7,8^. Moreover, when patients progress to severe ARDS, the mortality rate increases to more than 40%^9^. Therefore, the identification of key molecules in sepsis-associated ARDS and the search for markers for early diagnosis and possible therapeutic targets are important to reduce the mortality rate of sepsis-associated ARDS.

Pulmonary innate immunity plays a very important role in the pathogenesis of ARDS. Macrophages are an important component of pulmonary innate immunity. Macrophages can express pattern recognition receptors and thus recognize pathogen-associated molecular patterns (PAMPs) and damage-associated molecular patterns (DAMPs)^7,10^. Macrophages release proinflammatory mediators that induce the infiltration of neutrophils and other immune cells into the lungs. These immune cells further release inflammatory factors that initiate the innate immune response, causing damage to alveolar epithelial cells and pulmonary vascular endothelial cells, increased vascular permeability and the development of pulmonary oedema^11,12^. Macrophages usually have two polarization states: classically activated M1 macrophages or alternatively activated M2 macrophages. The balance of the M1/M2 phenotypes determines the different states of the organ in inflammation or injury^13^. In the acute exudative phase of ARDS, macrophages are predominantly M1-polarized and can release proinflammatory factors such as tumour necrosis factor alpha (TNF-α), interleukin 1 (IL-1), and reactive oxygen species (ROS), which induce a severe inflammatory response. In the late stage of ARDS, macrophages are mainly M2-polarized macrophages that can suppress the inflammatory response. However, excessive M2 polarization can lead to pathological fibroplasia and pulmonary fibrosis^14,15^. Therefore, macrophages have great research value in ARDS.

In recent years, although therapeutic agents for sepsis-induced ARDS have been investigated, effective biological targets for the treatment of ARDS have still not been identified. Previous reports have demonstrated the potential impact of genes on the treatment of ARDS^16^. With the development of bioinformatics, effective diagnostic and therapeutic targets for ARDS can be uncovered using bioinformatics techniques^17^. Machine learning is a branch of artificial intelligence that relates the problem of learning from data samples to the general concept of reasoning. Artificial neural network (ANN), random forest (RF), logistic regression, and support vector machine (SVM) methods are commonly used machine learning methods^18^. Among them, the RF method is an integrated approach that constructs a large number of decision trees for regression and classification tasks. The SVM method involves a supervised learning model that is very powerful in identifying subtle patterns in complex datasets^19^. Machine learning in conjunction with bioinformatics can transform biomedical big data into valuable knowledge and has been successfully applied to solve problems related to the fields of biology and medicine with good performance in terms of accuracy and speed^20,21^.

In this study, we retrieved and screened the sepsis-induced ARDS dataset from the Gene Expression Omnibus (GEO) database, screened for differentially expressed genes (DEGs) between sepsis-induced ARDS and normal groups, screened key genes using functional enrichment analysis, immune cell infiltration analysis, weighted gene correlation network analysis (WGCNA) and machine learning approaches and validated them in an external dataset. We explored the role of macrophage-related genes in the development of sepsis-induced ARDS and identified their molecular subtypes to expand the range of potential diagnostic biomarkers.

## Results

### Screening of differentially expressed genes in GSE32707

**Figure 1:**
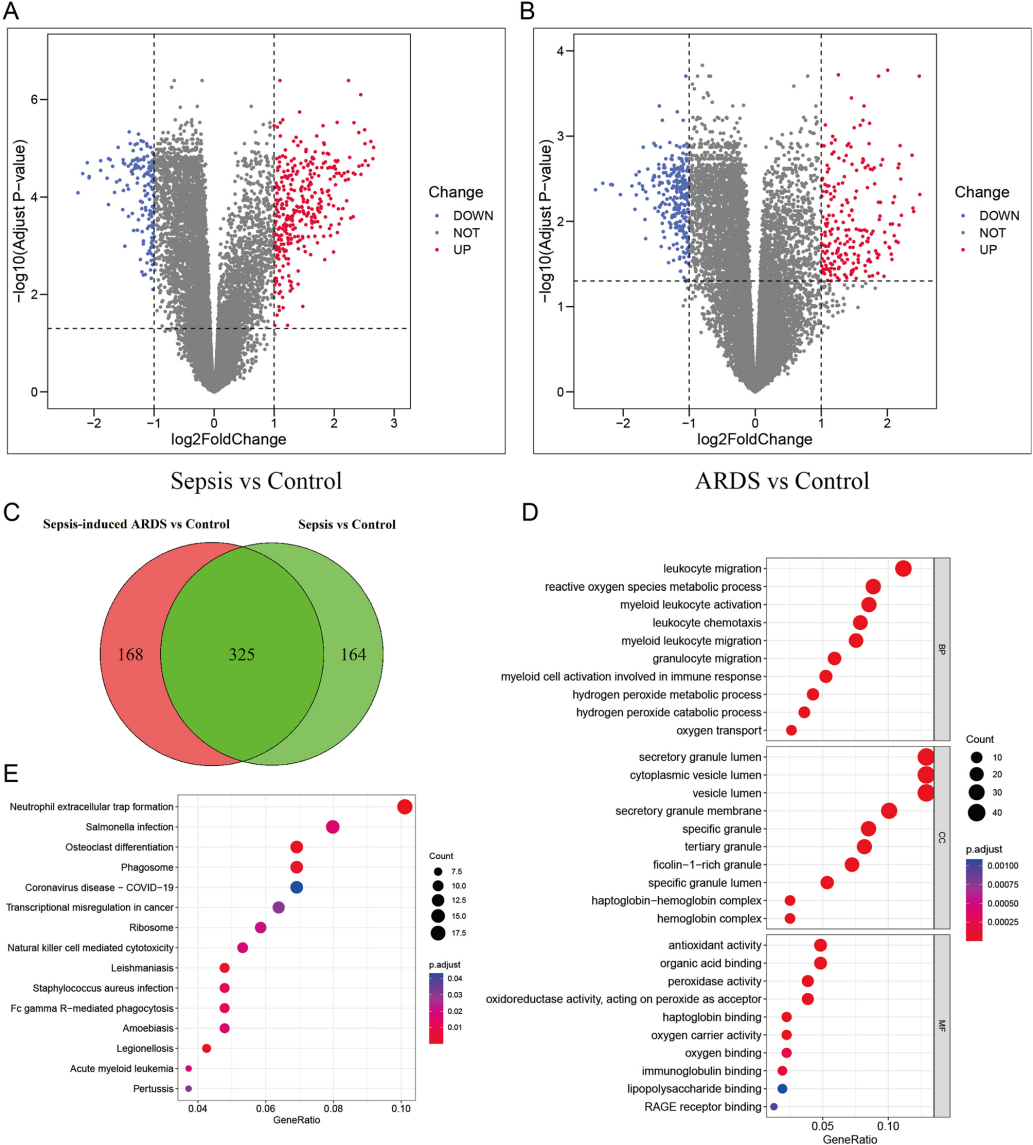
GSE32707 differential gene expression screening and enrichment analysis. (**A**) Volcano plot of differentially expressed genes between the sepsis group and control group in the GSE32707 dataset. (**B**) Volcano plot of differentially expressed genes between the sepsis-induced ARDS group and control group in the GSE32707 dataset. (**C**) Venn plot of differentially expressed genes between the sepsis-induced ARDS group and control group. (Software: R (4.0.2) version, R packet: VennDiagram (1.7.3). URL: https://cran.rstudio.com/web/packages/VennDiagram/index.html). D. GO enrichment analysis of differentially expressed genes. E. KEGG enrichment analysis of differentially expressed genes. ((D, E) Software: R (4.0.2) version, R packet: clusterProfiler (4.6.2). URL: https://bioconductor.org/packages/release/bioc/html/clusterProfiler.html).

According to the screening criteria of differentially expressed genes, there were 489 differentially expressed genes between the control and sepsis groups, of which 152 genes were downregulated in sepsis patients and 337 genes were upregulated in sepsis patients (Fig. 1A). In contrast, there were 493 differentially expressed genes between the control and sepsis-induced ARDS groups, of which 267 genes were downregulated in ARDS patients and 226 genes were upregulated in ARDS patients (Fig. 1B) (Supplementary Table S1). The differentially expressed genes between the two groups were intersected, and a total of 325 common differentially expressed genes were obtained (Fig. 1C).

### Enrichment analysis of differentially expressed genes

To understand the main functions of the 325 differentially expressed genes, Gene Ontology (GO) and Kyoto Encyclopedia of Genes and Genomes (KEGG) enrichment analyses were performed. The GO analysis suggested that the top five enriched biological processes (BPs) were leukocyte migration, reactive oxygen species metabolic process, myeloid leukocyte activation, hydrogen peroxide catabolic process and leukocyte chemotaxis. The top five enriched cellular components (CCs) were secretory granule lumen, cytoplasmic vesicle lumen, vesicle lumen, specific granule and tertiary granule. The top 5 enriched molecular functions (MFs) were antioxidant activity, haptoglobin binding, peroxidase activity, oxidoreductase activity, acting on peroxide as acceptor and oxygen carrier activity (Fig. 1D and Supplementary Table S2).

KEGG enrichment analysis suggested that the main enriched pathways were neutrophil extracellular trap formation, osteoclast differentiation, legionellosis, phagosome, leishmaniasis, Staphylococcus aureus infection and Fc gamma R-mediated phagocytosis (Fig. 1E and Supplementary Table S3). The GO and KEGG analysis results showed a significant correlation of differentially expressed genes with immune function and reactive oxygen species metabolism.

### Gene set variation analysis (GSVA)

To understand the differences in enrichment pathway levels between control, sepsis and sepsis-induced ARDS samples, GSVA was performed using the “c2.cp.kegg.v7.5.1.symbols.gmt” gene set as a reference.

The enrichment levels of cytokine-cytokine receptor interaction, cytosolic DNA sensing pathway, the JAK/STAT signalling pathway, natural killer cell-mediated cytotoxicity, Toll-like receptor signalling pathway, and primary immunodeficiency were significantly higher in sepsis patients than in controls (*P* < 0.05), whereas the enrichment levels of glycosylphosphatidylinositol (GPI) anchor biosynthesis, arginine and proline metabolism, butanoate metabolism, limonene and pinene degradation, histidine metabolism, and lysine degradation were significantly lower than those in controls (*P* < 0.05) (Supplementary Fig. S1).

The enrichment levels of cytokine-cytokine receptor interaction, glycerophospholipid metabolism, systemic lupus erythematosus, adipocytokine signalling pathway, type II diabetes mellitus, and nitrogen metabolism were significantly higher in the sepsis-induced ARDS group than in the control group (*P* < 0.05). The enrichment levels of the TGF beta signalling pathway, GPI anchor biosynthesis, spliceosome, tryptophan metabolism, basal transcription factors, and limonene and pinene degradation terms were significantly lower than those in the control group (*P* < 0.05). These results indicated that there were significant differences in the enrichment levels of immune- and metabolism-related pathways between the control, sepsis and sepsis-induced ARDS groups (Supplementary Fig. S2) (Supplementary Table S4).

### Immune cell infiltration analysis

**Figure 2:**
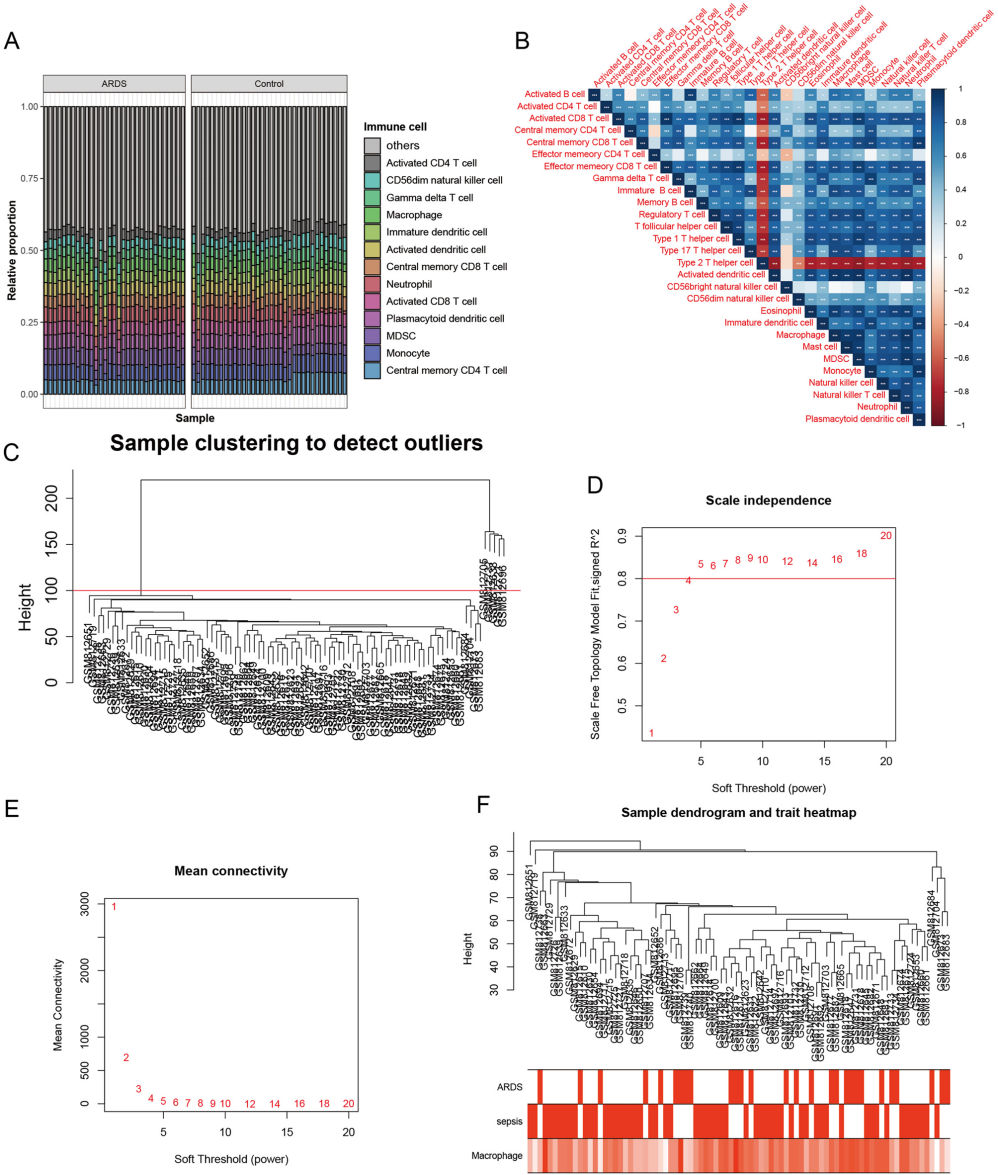
Immune infiltration analysis and WGCNA. (**A**) Graph of ssGSEA immune cell enrichment analysis in the GSE32707 dataset. (Software: R (4.0.2) version, R packet: GSVA (1.46.0). URL: https://github.com/rcastelo/GSVA). (**B**) ssGSEA immune cell level correlation. (Software: R (4.0.2) version, R packet: corrplot (0.92). URL: https://github.com/taiyun/corrplot). (**C**) Sample clustering dendrogram to detect outliers in WGCNA. D, E. Soft threshold selection process; F. Cluster dendrogram of samples using a Pearson correlation coefficient. The vertical axis indicates the relative distance between clusters. The smaller the height is, the more likely the samples are clustered together. The following feature heatmap shows the hierarchical clustering. ((**C**–**F**) Software: R (4.0.2) version, R packet: WGCNA (1.72–1) URL: http://horvath.genetics.ucla.edu/html/CoexpressionNetwork/Rpackages/WGCNA/).

As GO, KEGG and GSVA analyses suggested an immune correlation, immune infiltration analysis was performed to further understand the immune cell levels in the samples. Single-sample gene set enrichment analysis (ssGSEA) suggested that monocytes, neutrophils, macrophages and MDSCs were all present at high levels in ARDS patients (Fig. 2A and Supplementary Table S5). There was a good correlation between the levels of each immune cell (*P* < 0.05) (Fig. 2B).

### WGCNA

**Figure 3:**
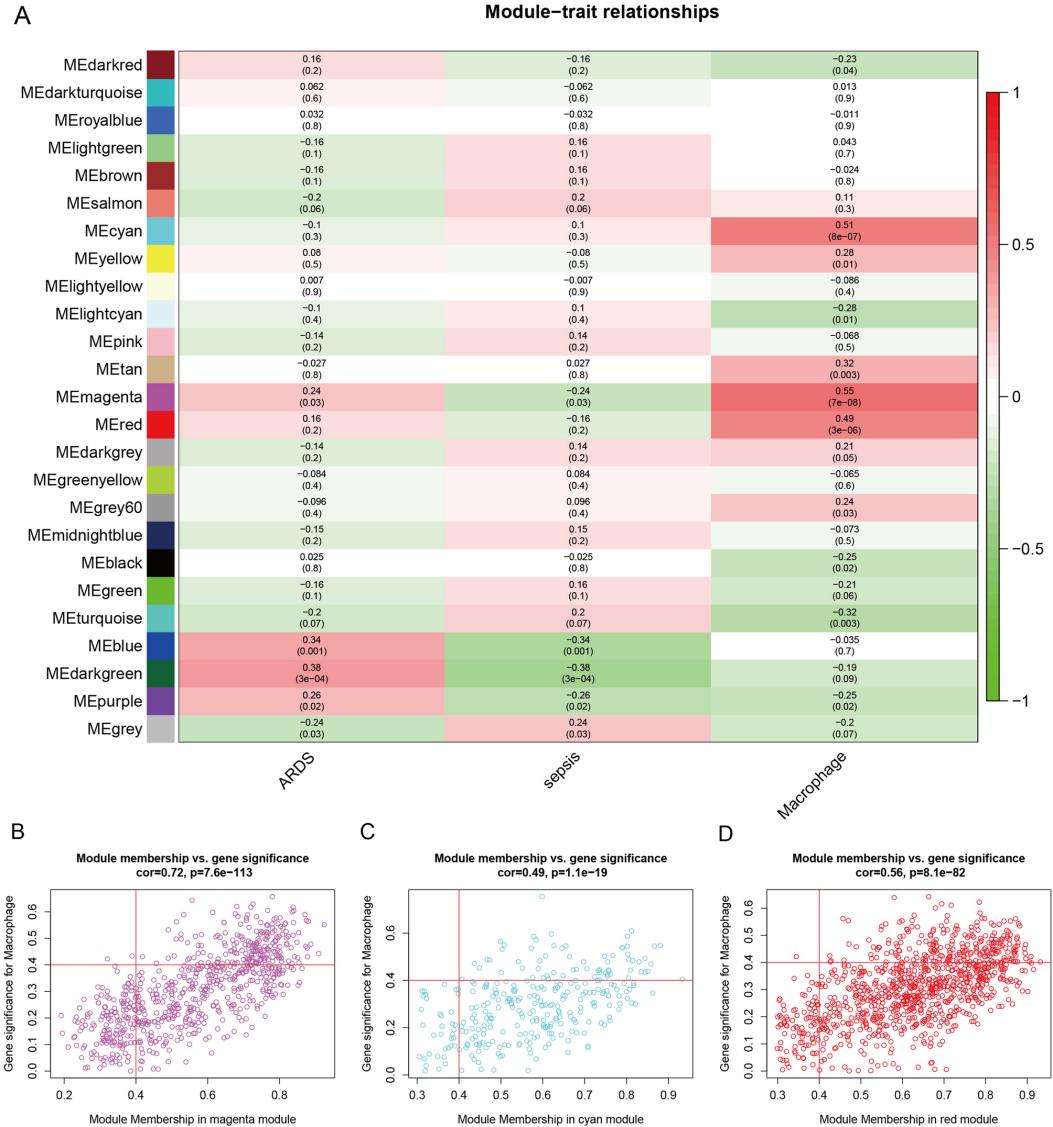
WGCNA-based screening of macrophage-related genes. (**A**) Heatmap of module features. The corresponding correlation coefficients and P values for each phenotype are shown. Correlation coefficients are indicated as follows: red rectangles indicate positive correlations; blue rectangles indicate negative correlations. (**B**) The correlation between magenta module membership and macrophage infiltration. (**C**) The correlation between cyan module membership and macrophage infiltration. (**D**) The correlation between red module membership and macrophage infiltration. (Software: R (4.0.2) version, R packet: WGCNA (1.72–1) URL: http://horvath.genetics.ucla.edu/html/CoexpressionNetwork/Rpackages/WGCNA/).

The sepsis and ARDS group samples in GSE32707 were subjected to WGCNA with a set cut-off height of 100, 5 samples were removed, and the remaining samples were subjected to subsequent analysis (Fig. 2C). According to the approximate scale-free topology criterion, a soft threshold of 5 was set to define the adjacency matrix (Fig. 2D,E). A clustering dendrogram (Fig. 2F) was constructed based on the matrix, and a total of 24 modules were obtained to distinguish different modules with different colours (Fig. 3A). The correlation analysis of each module with macrophages suggested that the magenta, cyan and red modules were significantly positively correlated with macrophages (Fig. 3B–D).

### Macrophage-related differentially expressed gene extraction, protein‒protein interaction (PPI) analysis and GO and KEGG enrichment analysis

**Figure 4:**
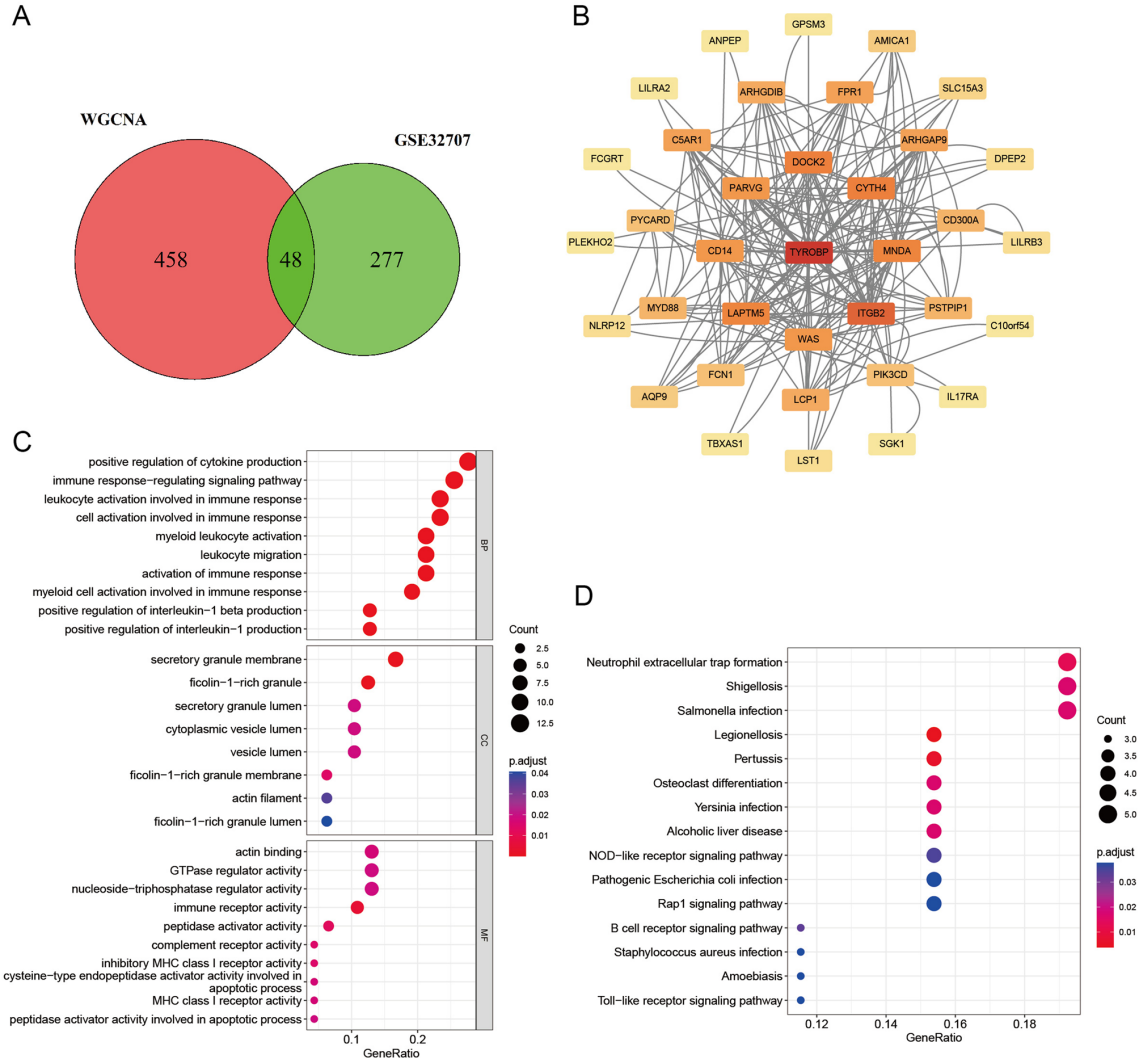
Screening of macrophage-related differentially expressed genes and PPI, GO, and KEGG enrichment analyses. (**A**) Venn diagram of macrophage-related genes screened in WGCNA with GSE32707 differentially expressed genes taken as intersection. (Software: R (4.0.2) version, R packet: VennDiagram (1.7.3). URL: https://cran.rstudio.com/web/packages/VennDiagram/index.html). (**B**) PPI diagram of macrophage-related differentially expressed genes. (**C**) GO enrichment analysis of macrophage-related differentially expressed genes. (**D**) KEGG enrichment analysis of macrophage-related differentially expressed gene. ((**C**, **D**) Software: R (4.0.2) version, R packet: clusterProfiler (4.6.2). URL: https://bioconductor.org/packages/release/bioc/html/clusterProfiler.html).

In the magenta, cyan and red modules, the correlation coefficients between genes and modules and the correlation between genes and macrophage levels were set to 0.4, resulting in the screening of 199 genes in the magenta module, 66 genes in the cyan module and 241 genes in the red module, for a total of 506 genes associated with macrophage infiltration level. These genes were intersected with the 325 differentially expressed genes screened in GSE327907 to obtain a total of 48 macrophage-related differentially expressed genes (Fig. 4A).

To understand the interaction relationship between genes, PPI analysis of macrophage-related differentially expressed genes was performed using the STRING database. The results suggested that TYROBP was the gene that interacted most with other genes, followed by ITGB2, CYTH4, DOCK2 and MNDA (Fig. 4B).

GO analysis suggests that the top five enriched BPs were myeloid cell activation involved in the immune response, leukocyte activation involved in the immune response, positive regulation of cytokine production, cell activation involved in the immune response, and myeloid leukocyte activation. The top 5 enriched CCs were secretory granule membrane, ficolin-1-rich granule, ficolin-1-rich granule membrane, secretory granule lumen and cytoplasmic vesicle lumen. The top 5 enriched MFs were immune receptor activity, peptidase activator activity, complement receptor activity, inhibitory MHC class I receptor activity and actin binding (Fig. 4C and Supplementary Table S6).

KEGG enrichment analysis suggests that the main enriched pathways are Legionellosis, Pertussis, Neutrophil extracellular trap formation, Osteoclast differentiation, Yersinia infection, Shigellosis, Alcoholic liver disease, Salmonella infection and B-cell receptor signalling pathway (Fig. 4D and Supplementary Table S7).

### Random forest plot and support vector machine (SVM) analyses

**Figure 5:**
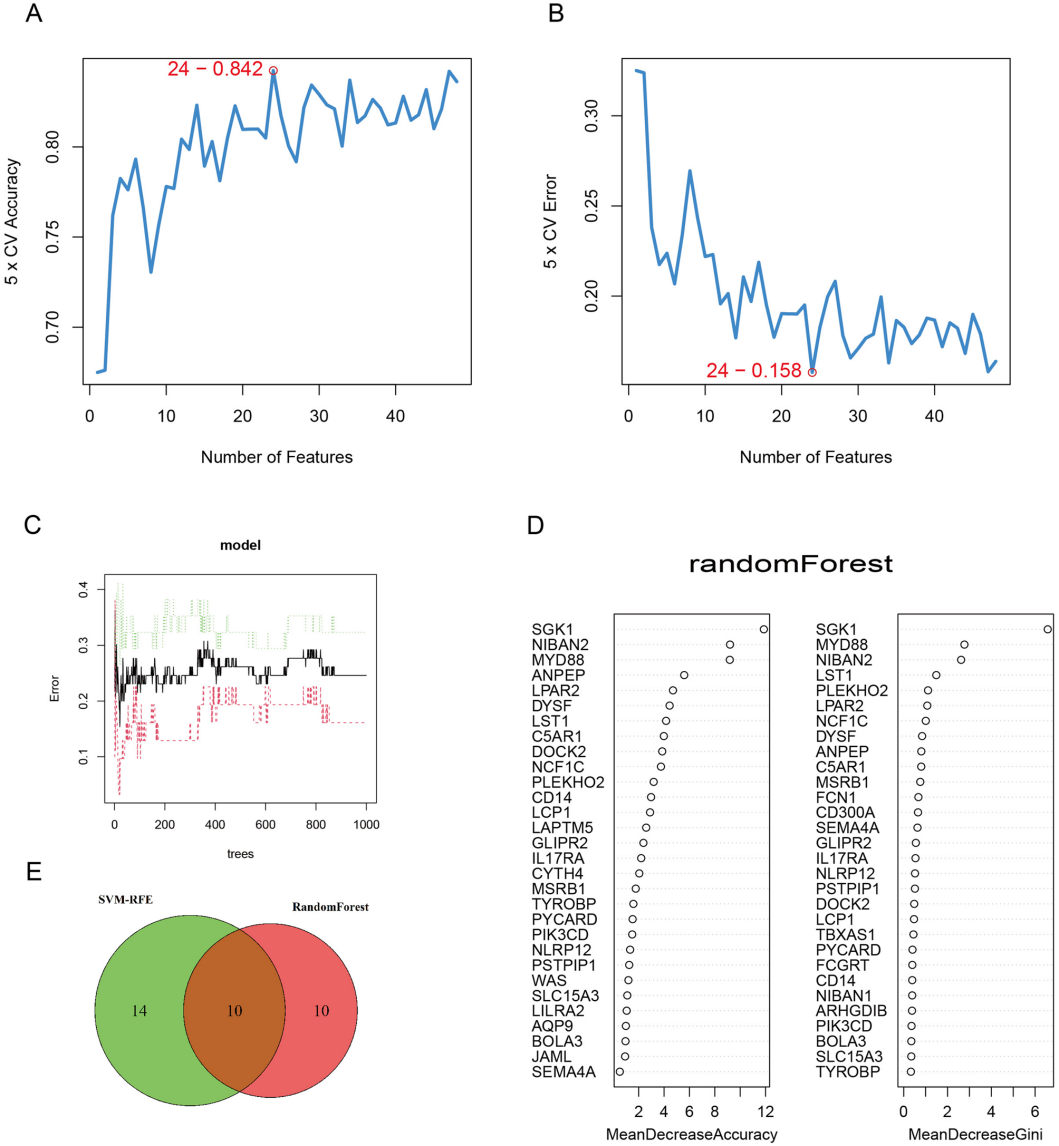
Random forest plot and SVM analysis. (**A**)–(**B**). SVM-RFE analysis plot; the horizontal axis represents the number of feature genes. The best fivefold cross-validation precision and error are 0.842 and 0.158, respectively. (Software: R (4.0.2) version, R packet: e1071 (1.7–13). URL: https://cran.rstudio.com/web/packages/e1071/index.html). (**C**) Random forest tree. (**D**) The precision and Gini coefficient of random forest plot analysis of macrophage-related differentially expressed genes to determine gene importance. ((**C**, **D**) Software: R (4.0.2) version, R packet: randomForestSRC (3.2.1). URL: https://www.randomforestsrc.org/https://ishwaran.org/). (**E**) SVM-RFE method and random forest plot method to screen genes in the intersection of the Venn diagram.

Random forest plot analysis and the SVM method were used to identify markers. The support vector machine recursive feature elimination (SVM-RFE) algorithm was used to screen the marker genes among the 48 genes, and a total of 24 genes were screened (Fig. 5A,B). The relative importance of the 48 genes was analysed using random forest plots, from which the top 20 genes in terms of relative importance were selected for subsequent analysis (Fig. 5C,D). Ten overlapping genes were finally identified between the two algorithms (Fig. 5E).

### Differential expression validation in the validation dataset

**Figure 6:**
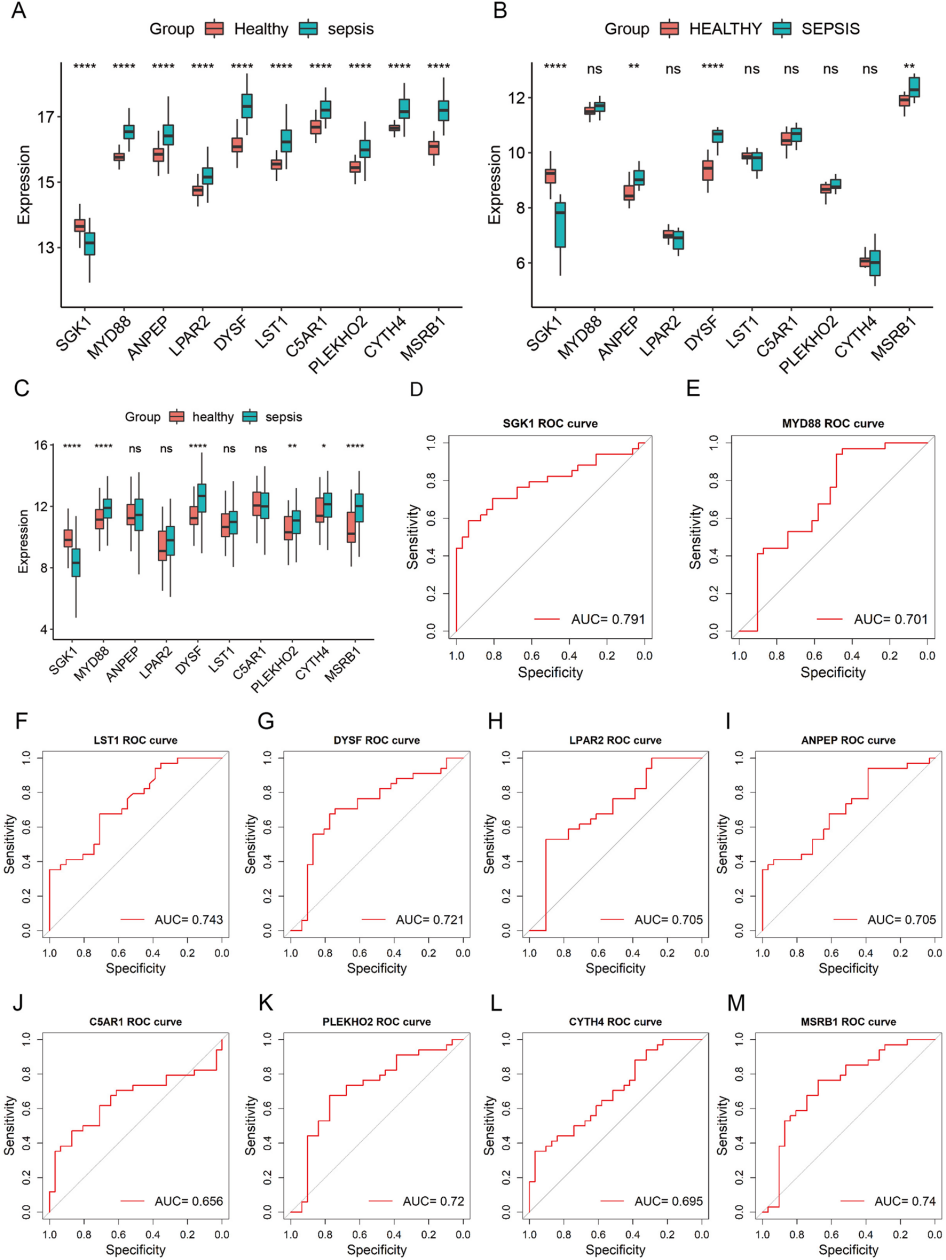
Gene expression validation. (**A**) Gene expression validation in the GSE154918 dataset. (**B**) Gene expression validation in the GSE28750 dataset. (**C**) Gene expression validation in the GSE185263 dataset. (Software: R (4.0.2) version, R packet: ggplot2 (3.4.1). URL: https://ggplot2.tidyverse.org/). (**D**)–(**M**). ROC analysis of the value of these genes in diabetes mellitus diagnosis. (Software: R (4.0.2) version, R packet: pROC (1.18.0). URL: http://expasy.org/tools/pROC/).

The differential expression analysis of the genes obtained after random forest plot and SVM analyses in the GSE154918, GSE28750, and GSE185263 datasets showed that, in GSE154918, the expression of all 10 genes was significantly different (*P* < 0.05) (Fig. 6A). In the GSE28750 dataset, the expression levels of SGK1, ANPEP, DYSF and MSRB1 were significantly different between groups (*P* < 0.05) (Fig. 6B). In the GSE185263 dataset, the expression levels of SGK1, MYD88, DYSF, PLEKHO2, CYTH4 and MSRB1 were significantly different between groups (*P* < 0.05) (Fig. 6C). Only SGK1, DYSF and MSRB1 showed differential expression in all three validation sets.

### ROC curve analysis of the diagnostic value of marker genes

To further understand the diagnostic value of marker genes in ARDS, ROC curve analysis was performed using samples from the control and sepsis-induced ARDS groups in the GSE32707 dataset as study samples. The results suggested that all 10 marker genes had a good predictive effect in terms of the diagnosis of ARDS, with an area under the curve greater than 0.65; SGK1 (AUC = 0.791) had the best diagnostic effect, followed by LST1 (AUC = 0.743), MSRB1 (AUC = 0.740) and DYSF (AUC = 0.721) (Fig. 6D–M). All three validated differentially expressed genes showed good diagnostic value for ARDS.

### Construction of the nomogram

**Figure 7:**
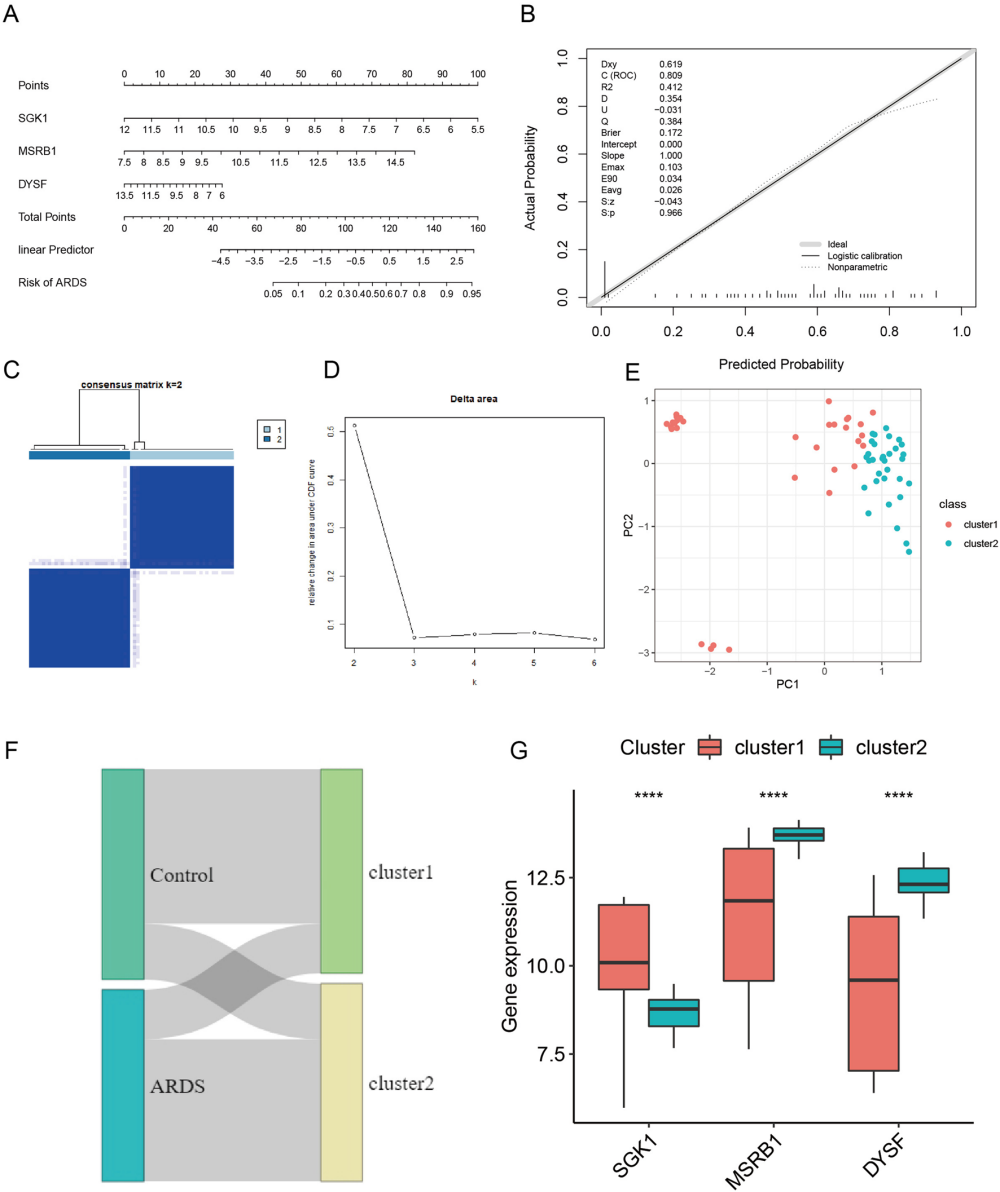
Nomogram and cluster analysis. (**A**) Nomogram plot. (**B**) Nomogram model prediction effect plot. (Software: R (4.0.2) version, R packet: rms (6.5–0). URL: https://hbiostat.org/R/rms/, https://github.com/harrelfe/rms). (**C**) Matrix heatmap of key gene clustering analysis. (**D**) Cluster analysis delta area plot. (**E**) PCA of different clusters after cluster analysis. (Software: R (4.0.2) version, R packet: ConsensusClusterPlus [1.62.0]. URL: https://bioconductor.org/packages/ConsensusClusterPlus/;). (**F**) Cluster analysis subtype grouping and sample consistency of sample diagnostic grouping Sankey plot. (**G**) Analysis of the expression differences of key genes in different clusters.

Based on the validation results of external datasets and ROC analysis, SGK1, DYSF and MSRB1 were used as the basic genes for subsequent analysis. To further understand the diagnostic value of SGK1, DYSF and MSRB1 in ARDS, nomogram analysis was performed, and a model was constructed. The calibration curves suggested that the nomogram model curves had high overlap with the ideal model, indicating that the nomogram model composed of three genes, SGK1, DYSF and MSRB1, showed good diagnostic prediction for ARDS. Moreover, the area under the curve (AUC) was 0.809, demonstrating that the prediction effect of the nomogram model was better than that of each of the three genes (SGK1, DYSF and MSRB1) alone (Fig. 7A,B).

### Clustering analysis

To understand the effect of SGK1, DYSF and MSRB1 on sample clustering, cluster analysis was performed. The results suggested that the samples could be divided into 2 clusters according to the expression values of SGK1, DYSF and MSRB1 (Fig. 7C,D). PCA showed that the cluster analysis had a good clustering effect (Fig. 7E). Sankey plots showed good agreement between cluster and disease grouping for cluster analysis, with Cluster 1 having a high proportion of samples in the control group and Cluster 2 having a high proportion of samples in the ARDS group (Fig. 7F). Wilcox analysis suggested that SGK1, DYSF and MSRB1 were differentially expressed in Cluster 1 and Cluster 2 (Fig. 7G) (*P* < 0.05), and the trend of difference was consistent with the trend of difference between the control and ARDS groups. These results further demonstrate the diagnostic value of SGK1, DYSF and MSRB1 for ARDS.

### Transcription factors and miRNA prediction of SGK1, DYSF and MSRB1

**Figure 8:**
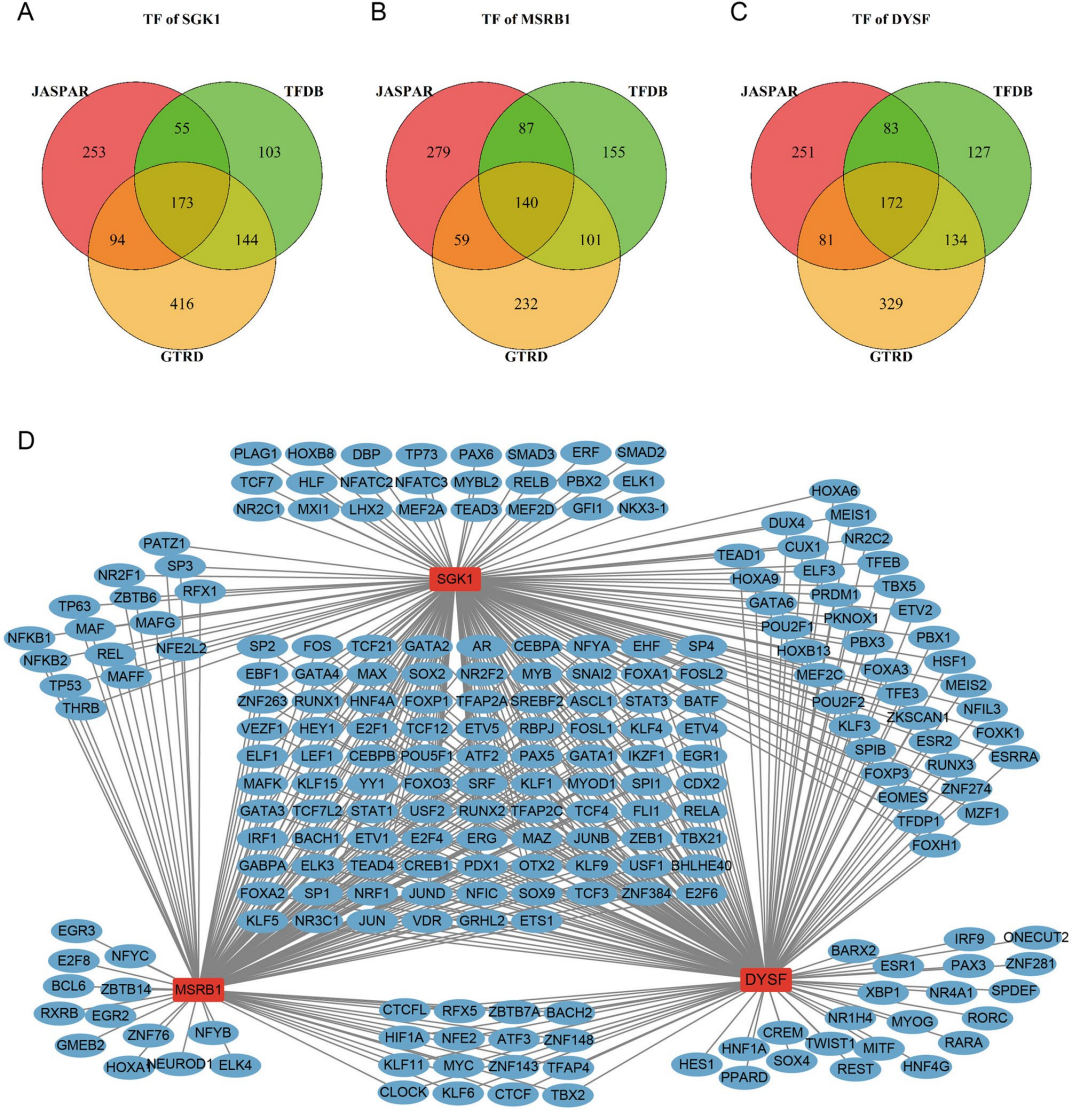
Transcription factor prediction. (**A**) Transcription factors of DYSF were predicted by three databases, as shown in Venn diagram. (**B**) Transcription factors of MSRB1 were predicted by three databases, as shown in Venn diagram. (**C**) Transcription factors of DYSF were predicted by three databases, as shown in Venn diagram. (Software: R (4.0.2) version, R packet: VennDiagram (1.7.3). URL: https://cran.rstudio.com/web/packages/VennDiagram/index.html). (**D**) Interaction of SGK1, MSRB1 and DYSF with predicted transcription factors.

Based on the prediction results in the JASPAR, HumanTFDB, and GTRD databases, a total of 173 transcription factors of SGK1 (Fig. 8A), 140 transcription factors of MSRB1 (Fig. 8B), and 172 transcription factors of DYSF (Fig. 8C,D) were obtained. Based on the prediction results in six databases, miRWalk, RNA22, RNAInter, TargetMiner, TargetScan and miRDB, a total of 28 miRNAs for SGK1 were obtained (see Supplementary Fig. S3A online). miRNAs for DYSF and MSRB1 were not predicted in the TargetMiner database, and the prediction results in the remaining five databases suggested that a total of 12 miRNAs of MSRB1 (see Supplementary Fig. S3B online) and 21 miRNAs of DYSF (see Supplementary Fig. S3C,D online) were obtained.

## Discussion

Sepsis affects more than 30 million people worldwide each year and is one of the leading causes of death in critically ill patients^22^. Sepsis-induced ARDS is a type of acute progressive respiratory failure caused by sepsis, and its pathogenesis is mostly focused on the inflammatory response, oxidative stress, and abnormal coagulation^23^. As innate immune cells, macrophages play a key role in the inflammatory response. It was found that secretory autophagy of alveolar macrophages (AMs) promotes the inflammatory response and lung injury through secreted IL-1β^24^. Impaired phagocytosis of alveolar macrophages in advanced sepsis further increases the severity of ARDS, and IFN-β treatment reverses the impairment of AM function induced by IL-10 and reduces the severity and mortality of ARDS in a dose-dependent manner^25^.

The results of the enrichment analysis in this study suggest significant enrichment of multiple pathways. Immune cells play a substantial role in the development and treatment of ARDS. Neutrophils play a major role in the process of leukocyte migration. Neutrophils are the first immune cells to migrate to the site of inflammation after stimulation by chemokines released from damaged lung tissue, and massive activation of neutrophils leads to peripheral tissue damage and lung dysfunction^26,27^. Eliav et al.^28^ found that the leukocyte migration inhibitor GT-73 significantly reduced the number of infiltrating leukocytes in LPS-induced ARDS in mice, decreased the levels of cytokines and was protective against ARDS. The reactive oxygen species-related pathway was also significantly enriched, and in ARDS, the production of reactive oxygen species (ROS) is detrimental to lung tissue, and ROS can damage lung endothelial cells and lead to impaired alveolar-capillary barrier function^29^. NF-κB is an important transcription factor that controls the release of proinflammatory mediators, and ROS can also cause activation of NF-κB, which can exacerbate inflammatory lung injury^30^. Studies have shown that suppressing the production of ROS is beneficial in reducing inflammatory lung injury^31^.

GSVA suggests significant differences in cytokine/cytokine receptor and Toll-like receptor pathway enrichment levels. In ARDS, multiple receptors of innate immune cells (macrophages, dendritic cells or monocytes), such as Toll-like receptors, recognize PAMPs and DAMPs to induce cytokine release syndrome^32^. It has been shown that the inflammatory response to LPS-induced ARDS can be attenuated by inhibiting TLR4 expression^33^. AM is a major source of cytokines and chemokines that initiate the immune response, and the overproduction of these proinflammatory cytokines (IL-1β, IL-6, TNF-α, IL-8, etc.) leads to the development of acute lung injury^34^. Additionally, AM is a major source of anti-inflammatory cytokines, such as IL-10 and TGF-β, which can suppress the inflammatory response to acute lung injury.

The three key genes screened in this study have been partially studied in lung injury and other diseases. SGK1 is a member of the protein kinase subfamily, a serine/threonine protein kinase with high homology to second messengers such as protein kinase B. As a hub for multiple signal transduction pathways and cellular phosphorylation events, SGK1 plays an important role in cell proliferation, ion channel regulation, signal transduction and other physiological processes and is thought to have an essential function in inflammation^35^. Studies have shown that SGK1 enhances the function of sodium channels to promote clearance of alveolar oedema fluid in a mouse model of lung injury^36,37^. Michalick et al. found that SGK1 may provide promising new targets for the prevention or treatment of ventilator-associated lung injury^38^. Xi^39^ et al. found that SGK1 exacerbates the inflammatory response by inducing macrophage activation and promoting the development of hypoxia-induced pulmonary hypertension in mice. Therefore, SGK1 was suggested as a possible target for the treatment of pulmonary hypertension. DYSF encodes dysferlin, a protein enriched with seven β-folded structural domains composed of C2, which mainly functions in calcium-dependent cell membrane regeneration and maintenance. Although dysferlin is mainly expressed in muscle tissue, expression can also be observed in nonskeletal muscle tissues and cells, such as blood monocytes and macrophages, which also express dysferlin^40^. DYSF was found to enhance phagocytosis, migration and adhesion of THP1 cells. In atherosclerotic disease, DYSF promoter methylation promotes its expression and promotes monocyte activation, further participating in the development of atherosclerotic cardiovascular disease^41^. MSRB1 is a member of the selenoprotein family and contains a selenocysteine residue at its catalytic site that specifically catalyses the free and protein-bound R-methionine sulfoxide to methionine. Studies have shown that MSRB1 is closely associated with diseases or disorders related to oxidative damage, such as ageing, neurodegenerative diseases and diabetes^42^. Lee^43^ et al. found that MSRB1 controls the immune response by promoting the expression of anti-inflammatory cytokines in macrophages. It was found^44^ that MSRB1 activates the STAT6 pathway in dendritic cells, which induces dendritic cell maturation and IL-12 production, promoting Th1 differentiation. In addition, MSRB1 promotes the differentiation of follicular helper T cells. This study reveals a role for the MSRB1 selenoprotein in adaptive immunity and indicates that targeting MSRB1 may have therapeutic potential in controlling immune responses. All three key genes are of great interest, as they can influence disease progression by affecting the function of macrophages. In this study, all three genes, SGK1, DYSF and MSRB1, showed good diagnostic effects. The nomogram model including all three genes further showed excellent predictive performance with an AUC value of 0.998. In addition, the levels of all three genes were assessed in an external validation cohort, and their relative expression trends were consistent.

The present study has several limitations. First, the data in this study were all from the GEO database, and further experimental evidence is needed to analyse the differential gene expression. Second, basic experiments are needed to investigate the mechanism of gene action in ARDS, and finally, more preclinical studies and prospective clinical trials are needed to validate our findings.

In conclusion, we used bioinformatics methods to screen three key macrophage-related genes, SGK1, DYSF and MSRB1, which have a good diagnostic effect on sepsis-induced ARDS. The nomogram model composed of these three genes also showed a good diagnostic effect, providing new targets for the early diagnosis of sepsis-induced ARDS. However, further experimental and clinical studies are needed to confirm these findings.

## Materials and methods

### Data acquisition

The GEO database (https://www.ncbi.nlm.nih.gov/geo/) was searched using the search formula “((Expression profiling by array[Filter]) AND Homo sapiens [Organism]) AND whole blood samples AND sepsis”. Two suitable datasets, GSE32707 and GSE28750, were obtained. The GEO database was searched using the search formula “((Expression profiling by high throughput sequencing [Filter]) AND Homo sapiens [Organism]) AND sepsis”. Two suitable datasets, GSE154918 and GSE185263, were obtained. The dataset includes clinical information and gene expression information of patients. The GSE32707 dataset, obtained using the GPL10558 platform, contains gene expression information of 144 samples of whole blood samples, including patients with sepsis, sepsis complicated by ARDS, control and systemic inflammation groups. GSE154918, GSE28750, and GSE185263 were used as external validation datasets for key gene expression. The GSE154918 dataset, obtained using the GPL20301 platform, includes 40 healthy samples and 53 sepsis samples. The GSE28750 dataset, obtained using the GPL570 platform, includes 20 healthy samples and 20 sepsis samples. The GSE185263 dataset, obtained using the GPL16791 platform, includes 44 healthy samples and 348 sepsis samples.

### Differentially expressed gene screening

The limma package in R language was used for differential expression analysis of genes. Gene expression values of all samples were normalized using the limma package^45^. The differentially expressed genes in GSE32707 were analysed between the control group and sepsis group and between the control group and sepsis-induced ARDS group. The screening criteria of differentially expressed genes were | log_2_FC |> 1 and adjusted *P* value < 0.05. Then, the intersection of two differentially expressed genes was taken using the Venn diagram.

### Enrichment analysis of differentially expressed genes

Gene Ontology (GO) analysis and Kyoto Encyclopedia of Genes and Genomes (KEGG) analysis of differentially expressed genes in the GSE32707 dataset were performed using the “clusterProfiler” package in R^46^. GO analysis consists of three components: BP, CC, and MF. *P* < 0.05 was recognized as a significant term/pathway.

### Gene set variation analysis (GSVA)

The GSVA method was used to evaluate the enrichment level of pathways between the GSE32707 dataset control group and sepsis group and the control group and sepsis-induced ARDS group^47^. The gene set “c2.cp.kegg.v7.5.1.symbols.gmt” downloaded from the MsigDB online database was used as a reference. The R package “GSVA” was used to calculate the enrichment levels of pathways associated with each sample in the GSE32707 dataset, and then the "limma" package was used to analyse the differences in pathway enrichment levels between the control and sepsis groups and between the control and sepsis-induced ARDS groups. The screening conditions for significant differences were |t|> 2 and *P* value < 0.05.

### Immune cell infiltration analysis

Based on the gene expression level of each sample in the GSE32707 dataset, the enrichment level of 28 immune cells in each sample in the dataset was analysed using the ssGSEA method in R language. The corrplot package was used to analyse the relationship between the individual immune cell levels.

### Weighted gene correlation network analysis (WGCNA)

WGCNA was performed on all samples using the WGCNA package based on the expression levels of the sample genes^48^. Analysis of gene modules associated with macrophage levels in patients with sepsis and sepsis-induced ARDS. Cluster analysis was performed on all samples, and abnormal samples were excluded. A soft threshold (β) for network construction in the WGCNA algorithm was selected, the “min Module Size” was set to 100, and the coexpressed genes were assigned to modules by the dynamic minimal tree cutting algorithm. By analysing the correlation between modules and phenotypes, gene coexpression modules significantly associated with macrophage levels were identified.

### GO and KEGG enrichment analysis of macrophage-related differential genes

The macrophage-related key module genes in WGCNA were intersected with the obtained GSE32707 differentially expressed genes to obtain the differentially expressed genes related to the macrophage level. Using the "cluster Profiler" package in the R language, GO analysis and KEGG analysis were performed on the differentially expressed genes related to macrophage level to obtain the major enriched pathways.

### Construction of PPI networks

The STRING database was used to analyse protein interactions with differentially expressed genes associated with the macrophage level, and the minimum interaction score required for PPI was set at 0.4 (medium confidence level)^49^. The results were imported into Cytoscape software and visualized using Cytoscape.

### Random forest graph analysis and support vector machine analysis

Macrophage-associated differentially expressed genes were rescreened using random forest graph analysis^50^ and SVM analysis^51^ methods to discriminate the most valuable genes. The best genes were selected from the metadata cohort using the recursive feature elimination method to avoid overfitting. Therefore, SVM-RFE (support vector machine recursive feature elimination) was used to discover the set of genes with the greatest discriminatory power. Random forest plots were also used to analyse the relative importance of individual genes in macrophage-associated differential genes. The genes in the intersection of the prediction results of the two methods were selected as the target genes.

### Differential expression validation in the validation dataset

The GSE154918, GSE28750, and GSE185263 datasets were used to validate the differential expression of the genes obtained after the random forest plot and SVM analysis. The Wilcox method was used to analyse the differential expression of genes in each dataset.

### Receiver operating characteristic (ROC) curve analysis of the diagnostic value of key genes

The pROC package in R language was used to analyse the diagnostic value of gene expression levels for ARDS after screening by random forest plot and SVM analysis^52^. Samples from the control and sepsis-induced ARDS groups in the GSE32707 dataset were used as study samples, and the pROC package was used to calculate area under curve (AUC) values to show their diagnostic value. The ROC curve results were visualized using the 'plot.roc' function.

### Nomogram diagram

The nomogram model was constructed using the rms package. Logistic regression analysis related to ARDS diagnosis was first performed using genes that had been screened and verified for expression differences, and then the nomogram model was constructed and visualized based on the results of the logistic regression analysis. The scoring criteria were developed based on the magnitude of the model regression coefficients, and by assigning a score to each gene for each value taken, the total score for each patient could be calculated. Then, the probability of ARDS occurrence for each patient was calculated based on the conversion function between the score and the probability of outcome occurrence. Finally, the model ROC curve area was calculated, and the calibration curve was plotted and evaluated (calibration degree, U test).

### Clustering analysis

Unsupervised cluster analysis was performed in the samples of the control and ARDS groups in the GSE32707 dataset based on the genes verified by expression differences using the Consensus Cluster Plus package in the R language^53^. PCA was used to analyse the effect of cluster analysis, and the network D3 package was used to perform Sankey map analysis to analyse the consistency of grouping of cluster analysis with sample grouping. Wilcox test was used to analyse the differences in gene expression between different clustered groupings.

### Transcription factors and miRNA prediction of key genes

The promoter sequences of the core genes (including the transcription start site up to 2000 bp upstream of it) were obtained from the NCBI database (https://www.ncbi.nlm.nih.gov/gene/). After that, transcription factor prediction was performed in the JASPAR database (https://jaspar.genereg.net/), HumanTFDB database (http://bioinfo.life.hust.edu.cn/HumanTFDB#!/) and GTRD database (http://gtrd.biouml.org/#!) based on the obtained promoter sequences. The transcription factors obtained from the three databases were intersected to obtain transcription factors that existed in all three databases at the same time and then visualized using Cytoscape software. miRNAs of key genes were predicted in the miRWalk, RNA22, RNAInter, TargetMiner, TargetScan and miRDB databases, and then the miRNAs in each database were overlapped to identify the intersecting miRNAs; the Venn diagram was visualized using Cytoscape software.

## Supplementary Information

Supplementary Figures.

Supplementary Table S1.

Supplementary Table S2.

Supplementary Table S3.

Supplementary Table S4.

Supplementary Table S5.

Supplementary Table S6.

Supplementary Table S7.

## Data Availability

The datasets analysed during the current research are all available in the GEO database (GSE32707, https://www.ncbi.nlm.nih.gov/geo/query/acc.cgi?acc=GSE32707; GSE154918, https://www.ncbi.nlm.nih.gov/geo/query/acc.cgi?acc=GSE154918; GSE28750, https://www.ncbi.nlm.nih.gov/geo/query/acc.cgi?acc=GSE28750; GSE185263,https://www.ncbi.nlm.nih.gov/geo/query/acc.cgi?acc=GSE185263) and MsigDB (http://www.gsea-msigdb.org/gsea/index.jsp).

## References

[1] David S, Brunkhorst F (2017). Sepsis-3: What has been confirmed in therapy?. Der Internist.

[2] Chiu C, Legrand M (2021). Epidemiology of sepsis and septic shock. Curr. Opin. Anaesthesiol..

[3] L’Heureux M, Sternberg M, Brath L, Turlington J, Kashiouris M (2020). Sepsis-induced cardiomyopathy: A comprehensive review. Curr. Cardiol. Rep..

[4] Ren C, Yao R, Zhang H, Feng Y, Yao Y (2020). Sepsis-associated encephalopathy: A vicious cycle of immunosuppression. J. Neuroinflamm..

[5] Petejova N (2020). Acute kidney injury in septic patients treated by selected nephrotoxic antibiotic agents-pathophysiology and biomarkers-a review. Int. J. Mol. Sci..

[6] Matthay M (2019). Acute respiratory distress syndrome. Nat. Rev. Dis. Primers..

[7] Englert J, Bobba C, Baron R (2019). Integrating molecular pathogenesis and clinical translation in sepsis-induced acute respiratory distress syndrome. JCI Insight.

[8] Bellani G (2016). Epidemiology, patterns of care, and mortality for patients with acute respiratory distress syndrome in intensive care units in 50 countries. JAMA.

[9] Pan C, Liu L, Xie J, Qiu H (2018). Acute respiratory distress syndrome: Challenge for diagnosis and therapy. Chin. Med. J..

[10] Kaku S (2020). Acute respiratory distress syndrome: Etiology, pathogenesis, and summary on management. J. Intensive Care Med..

[11] Kumar V (2020). Pulmonary innate immune response determines the outcome of inflammation during pneumonia and sepsis-associated acute lung injury. Front. Immunol..

[12] Dang W (2022). The role of lung macrophages in acute respiratory distress syndrome. Inflamm Res. Off. J. Eur. Histamine Res. Soc..

[13] Locati M, Curtale G, Mantovani A (2020). Diversity, mechanisms, and significance of macrophage plasticity. Annu. Rev. Pathol..

[14] Chen X (2020). Macrophage polarization and its role in the pathogenesis of acute lung injury/acute respiratory distress syndrome. Inflamm. Res. Off. J. Eur. Histamine Res. Soc..

[15] Shapouri-Moghaddam A (2018). Macrophage plasticity, polarization, and function in health and disease. J. Cell. Physiol..

[16] Reilly J, Christie J, Meyer N (2017). Fifty years of research in ARDS. Genomic contributions and opportunities. Am. J. Respir. Crit. Care Med..

[17] Sun M, Yang Q, Hu C, Zhang H, Xing L (2022). Identification and validation of autophagy-related genes in sepsis-induced acute respiratory distress syndrome and immune infiltration. J. Inflamm. Res..

[18] Giassa I, Alexiou P (2021). Bioinformatics and machine learning approaches to understand the regulation of mobile genetic elements. Biology.

[19] Huang S (2018). Applications of support vector machine (SVM) learning in cancer genomics. Cancer Genom. Proteomics.

[20] Libbrecht M, Noble W (2015). Machine learning applications in genetics and genomics. Nat. Rev. Genet..

[21] Deo R (2015). Machine learning in medicine. Circulation.

[22] Huang M, Cai S, Su J (2019). The pathogenesis of sepsis and potential therapeutic targets. Int. J. Mol. Sci..

[23] Huppert L, Matthay M, Ware L (2019). Pathogenesis of acute respiratory distress syndrome. Semin. Respir. Crit. Care Med..

[24] Xu X (2022). Secretory autophagosomes from alveolar macrophages exacerbate acute respiratory distress syndrome by releasing IL-1β. J. Inflamm. Res..

[25] Hiruma T (2018). IFN-β improves sepsis-related alveolar macrophage dysfunction and postseptic acute respiratory distress syndrome-related mortality. Am. J. Respir. Cell Mol. Biol..

[26] Aulakh G (2018). Neutrophils in the lung: “The first responders”. Cell Tissue Res..

[27] Yang S, Tsai Y, Pan Y, Hwang T (2021). Understanding the role of neutrophils in acute respiratory distress syndrome. Biomed. J..

[28] Qu M (2022). Neutrophil extracellular traps-triggered impaired autophagic flux via METTL3 underlies sepsis-associated acute lung injury. Cell Death Discov..

[29] Liu X (2018). Neferine protects endothelial glycocalyx via mitochondrial ROS in lipopolysaccharide-induced acute respiratory distress syndrome. Front. Physiol..

[30] Jiang K (2018). Barbaloin protects against lipopolysaccharide (LPS)-induced acute lung injury by inhibiting the ROS-mediated PI3K/AKT/NF-κB pathway. Int. Immunopharmacol..

[31] Wagner J (2018). Sevoflurane posttreatment prevents oxidative and inflammatory injury in ventilator-induced lung injury. PLoS ONE.

[32] Root-Bernstein R (2021). Innate receptor activation patterns involving TLR and NLR synergisms in COVID-19, ALI/ARDS and sepsis cytokine storms: A review and model making novel predictions and therapeutic suggestions. Int. J. Mol. Sci..

[33] Tang S (2019). Pre-treatment with ten-minute carbon dioxide inhalation prevents lipopolysaccharide-induced lung injury in mice via down-regulation of toll-like receptor 4 expression. Int. J. Mol. Sci..

[34] Bissonnette E, Lauzon-Joset J, Debley J, Ziegler S (2020). Cross-talk between alveolar macrophages and lung epithelial cells is essential to maintain lung homeostasis. Front. Immunol..

[35] Lu R (2022). SGK1, a critical regulator of immune modulation and fibrosis and a potential therapeutic target in chronic graft-versus-host disease. Front. Immunol..

[36] Fei X (2021). Aldosterone alleviates lipopolysaccharide-induced acute lung injury by regulating epithelial sodium channel through PI3K/Akt/SGK1 signaling pathway. Mol. Cell. Probes.

[37] Li J (2020). Melatonin attenuates sepsis-induced acute lung injury through improvement of epithelial sodium channel-mediated alveolar fluid clearance via activation of SIRT1/SGK1/Nedd4-2 signaling pathway. Front. Pharmacol..

[38] Michalick L (2017). Transient receptor potential vanilloid 4 and serum glucocorticoid-regulated kinase 1 are critical mediators of lung injury in overventilated mice in vivo. Anesthesiology.

[39] Xi X (2019). SGK1 mediates hypoxic pulmonary hypertension through promoting macrophage infiltration and activation. Anal. Cell. Pathol. (Amst.).

[40] White Z, Milad N, Sellers S, Bernatchez P (2021). Effect of dysferlin deficiency on atherosclerosis and plasma lipoprotein composition under normal and hyperlipidemic conditions. Front. Physiol..

[41] Zhang X (2022). DYSF promotes monocyte activation in atherosclerotic cardiovascular disease as a DNA methylation-driven gene. Transl. Res. J. Lab. Clin. Med..

[42] Tarrago L (2022). The selenoprotein methionine sulfoxide reductase B1 (MSRB1). Free Radical Biol. Med..

[43] Lee B (2017). Selenoprotein MsrB1 promotes anti-inflammatory cytokine gene expression in macrophages and controls immune response in vivo. Sci. Rep..

[44] Lee H (2020). The selenoprotein MsrB1 instructs dendritic cells to induce T-Helper 1 immune responses. Antioxidants (Basel Switz.).

[45] Ritchie M (2015). limma powers differential expression analyses for RNA-sequencing and microarray studies. Nucleic Acids Res..

[46] Yu G, Wang L, Han Y, He Q (2012). clusterProfiler: An R package for comparing biological themes among gene clusters. OMICS.

[47] Hänzelmann S, Castelo R, Guinney J (2013). GSVA: Gene set variation analysis for microarray and RNA-seq data. BMC Bioinform..

[48] Langfelder P, Horvath S (2008). WGCNA: An R package for weighted correlation network analysis. BMC Bioinform..

[49] Szklarczyk D (2017). The STRING database in 2017: Quality-controlled protein-protein association networks, made broadly accessible. Nucleic Acids Res..

[50] Taylor J (2011). Random survival forests. J. Thorac. Oncol. Off. Publ. Int. Assoc. Study Lung Cancer.

[51] Liu Q (2011). Gene selection and classification for cancer microarray data based on machine learning and similarity measures. BMC Genom..

[52] Robin X (2011). pROC: An open-source package for R and S+ to analyze and compare ROC curves. BMC Bioinform..

[53] Wilkerson M, Hayes D (2010). ConsensusClusterPlus: A class discovery tool with confidence assessments and item tracking. Bioinformatics (Oxf. Engl.).

