# Supplementary material for: Identification of macrophage-related genes in sepsis-induced ARDS using bioinformatics and machine learning
Source: Sci Rep. 2023 Jun 19;13:9876. doi: 10.1038/s41598-023-37162-5 (PMC10279743; doi:10.1038/s41598-023-37162-5)
Supplement: Supplementary file 1 — Supplementary Figures. [file 41598_2023_37162_MOESM1_ESM.pdf]

Supplementary Figures：

Identification of macrophage-related genes in sepsis-induced ARDS using bioinformatics and machine learning

Qiuyue Li[1,†], Hongyu Zheng[2,†], Bing Chen[1,*]

1 Department of Emergency Medicine, The Second Hospital of Tianjin Medical University, No.23, Pingjiang Road, Hexi District, 300211, Tianjin, China

2 Department of Maxillofacial Surgery, The First Affiliated Hospital of Chongqing Medical University, No.1, Youyi Road, Yuzhong District,400016, Chongqing, China

† These authors have contributed equally to this work and share first authorship.

*** Corresponding author**

Correspondence: Bing Chen, chenbtmu@hotmail.com

Figure legend:

Supplementary Figure 1. GSVA enrichment pathway differences between the sepsis group and the control group in GSE32707.

Supplementary Figure 2. Differences in enriched pathways according to GSVA between the sepsis-induced ARDS group and the control group in GSE32707.

Supplementary Figure 3. miRNA prediction. A. Database prediction of SGK1 target miRNAs in the intersection of the Venn diagram. B. Database prediction of MSRB1 target miRNAs in the intersection of the Venn diagram. C. Database prediction of DYSF target miRNAs in the intersection of the Venn diagram. D. Correlation diagram between SGK1, MSRB1, DYSF and miRNAs.

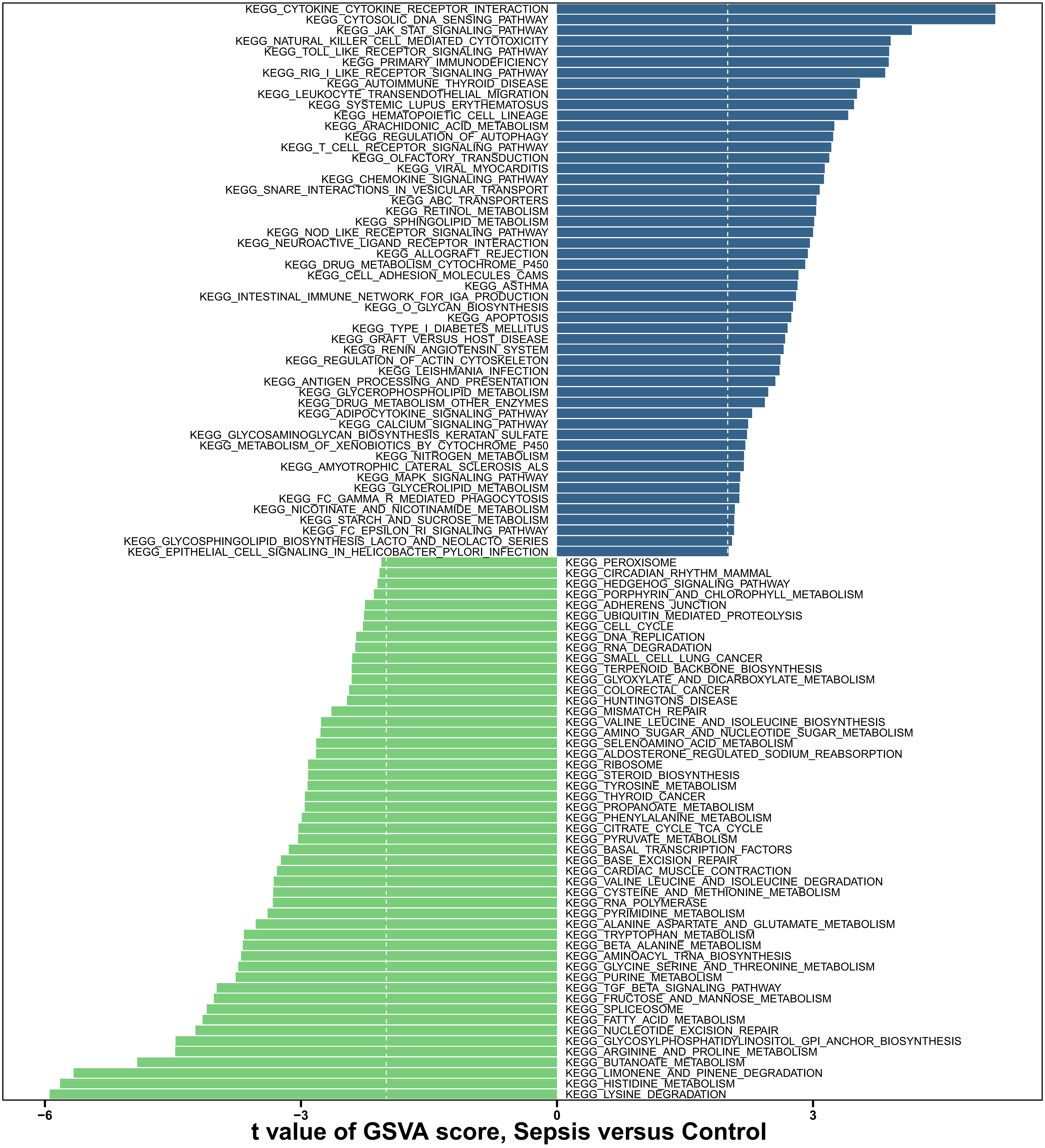
KEGG_CYTOKINE_CYTOKINE_RECEPTOR_INTERACTION
KEGG_CYTOSOLIC_DNA_SENSING_PATHWAY
KEGG_JAK_STAT_SIGNALING_PATHWAY
KEGG_NATURAL_KILLER_CELL_MEDIATED_CYTOTOXICITY
KEGG_TOLL_LIKE_RECEPTOR_SIGNALING_PATHWAY
KEGG_PRIMARY_IMMUNODEFICIENCY
KEGG_RIG_I_LIKE_RECEPTOR_SIGNALING_PATHWAY
KEGG_AUTOIMMUNE_THYROID_DISEASE
KEGG_LEUKOCYTE_TRANSENDOTHELIAL_MIGRATION
KEGG_SYSTEMIC_LUPUS_ERYTHEMATOSUS
KEGG_HEMATOPOIETIC_CELL_LINEAGE
KEGG_ARACHIDONIC_ACID_METABOLISM
KEGG_REGULATION_OF_AUTOPHAGY
KEGG_T_CELL_RECEPTOR_SIGNALING_PATHWAY
KEGG_OLFACTORY_TRANSDUCTION
KEGG_VIRAL_MYOCARDITIS
KEGG_CHEMOKINE_SIGNALING_PATHWAY
KEGG_SNARE_INTERACTIONS_IN_VESICULAR_TRANSPORT
KEGG_ABC_TRANSPORTERS
KEGG_RETINOL_METABOLISM
KEGG_SPHINGOLIPID_METABOLISM
KEGG_NOD_LIKE_RECEPTOR_SIGNALING_PATHWAY
KEGG_NEUROACTIVE_LIGAND_RECEPTOR_INTERACTION
KEGG_ALLOGRAFT_REJECTION
KEGG_DRUG_METABOLISM_CYTOCHROME_P450
KEGG_CELL_ADHESION_MOLECULES_CAMS
KEGG_ASTHMA
KEGG_INTESTINAL_IMMUNE_NETWORK_FOR_IGA_PRODUCTION
KEGG_O_GLYCAN_BIOSYNTHESIS
KEGG_APOPTOSIS
KEGG_TYPE_I_DIABETES_MELLITUS
KEGG_GRAFT_VERSUS_HOST_DISEASE
KEGG_RENIN_ANGIOTENSIN_SYSTEM
KEGG_REGULATION_OF_ACTIN_CYTOSKELETON
KEGG_LEISHMANIA_INFECTION
KEGG_ANTIGEN_PROCESSING_AND_PRESENTATION
KEGG_GLYCEROPHOSPHOLIPID_METABOLISM
KEGG_DRUG_METABOLISM_OTHER_ENZYMES
KEGG_ADIPOCYTOKINE_SIGNALING_PATHWAY
KEGG_CALCIUM_SIGNALING_PATHWAY
KEGG_GLYCOSAMINOGLYCAN_BIOSYNTHESIS_KERATAN_SULFATE
KEGG_METABOLISM_OF_XENOBIOTICS_BY_CYTOCHROME_P450
KEGG_NITROGEN_METABOLISM
KEGG_AMYOTROPHIC_LATERAL_SCLEROSIS_ALS
KEGG_MAPK_SIGNALING_PATHWAY
KEGG_GLYCEROLIPID_METABOLISM
KEGG_FC_GAMMA_R_MEDIATED_PHAGOCYTOSIS
KEGG_NICOTINATE_AND_NICOTINAMIDE_METABOLISM
KEGG_STARCH_AND_SUCROSE_METABOLISM
KEGG_FC_EPSILON_RI_SIGNALING_PATHWAY
KEGG_GLYCOSPHINGOLIPID_BIOSYNTHESIS_LACTO_AND_NEOLACTO_SERIES
KEGG_EPITHELIAL_CELL_SIGNALING_IN_HELICOBACTER_PYLORI_INFECTION
KEGG_PEROXISOME
KEGG_CIRCADIAN_RHYTHM_MAMMAL
KEGG_HEDGEHOG_SIGNALING_PATHWAY
KEGG_PORPHYRIN_AND_CHLOROPHYLL_METABOLISM
KEGG_ADHERENS_JUNCTION
KEGG_UBIQUITIN_MEDIATED_PROTEOLYSIS
KEGG_CELL_CYCLE
KEGG_DNA_REPLICATION
KEGG_RNA_DEGRADATION
KEGG_SMALL_CELL_LUNG_CANCER
KEGG_TERPENOID_BACKBONE_BIOSYNTHESIS
KEGG_GLYOXYLATE_AND_DICARBOXYLATE_METABOLISM
KEGG_COLORECTAL_CANCER
KEGG_HUNTINGTONS_DISEASE
KEGG_MISMATCH_REPAIR
KEGG_VALINE_LEUCINE_AND_ISOLEUCINE_BIOSYNTHESIS
KEGG_AMINO_SUGAR_AND_NUCLEOTIDE_SUGAR_METABOLISM
KEGG_SELENOAMINO_ACID_METABOLISM
KEGG_ALDOSTERONE_REGULATED_SODIUM_REABSORPTION
KEGG_RIBOSOME
KEGG_STEROID_BIOSYNTHESIS
KEGG_TYROSINE_METABOLISM
KEGG_THYROID_CANCER
KEGG_PROPANOATE_METABOLISM
KEGG_PHENYLALANINE_METABOLISM
KEGG_CITRATE_CYCLE_TCA_CYCLE
KEGG_PYRUVATE_METABOLISM
KEGG_BASAL_TRANSCRIPTION_FACTORS
KEGG_BASE_EXCISION_REPAIR
KEGG_CARDIAC_MUSCLE_CONTRACTION
KEGG_VALINE_LEUCINE_AND_ISOLEUCINE_DEGRADATION
KEGG_CYSTEINE_AND_METHIONINE_METABOLISM
KEGG_RNA_POLYMERASE
KEGG_PYRIMIDINE_METABOLISM
KEGG_ALANINE_ASPARTATE_AND_GLUTAMATE_METABOLISM
KEGG_TRYPTOPHAN_METABOLISM
KEGG_BETA_ALANINE_METABOLISM
KEGG_AMINOACYL_TRNA_BIOSYNTHESIS
KEGG_GLYCINE_SERINE_AND_THREONINE_METABOLISM
KEGG_PURINE_METABOLISM
KEGG_TGF_BETA_SIGNALING_PATHWAY
KEGG_FRUCTOSE_AND_MANNOSE_METABOLISM
KEGG_SPLICEOSOME
KEGG_FATTY_ACID_METABOLISM
KEGG_NUCLEOTIDE_EXCISION_REPAIR
KEGG_GLYCOSYLPHOSPHATIDYLINOSITOL_GPI_ANCHOR_BIOSYNTHESIS
KEGG_ARGININE_AND_PROLINE_METABOLISM
KEGG_BUTANOATE_METABOLISM
KEGG_LIMONENE_AND_PINENE_DEGRADATION
KEGG_HISTIDINE_METABOLISM
KEGG_LYSINE_DEGRADATION
−6
−3
0
3
t value of GSVA score, Sepsis versus Control

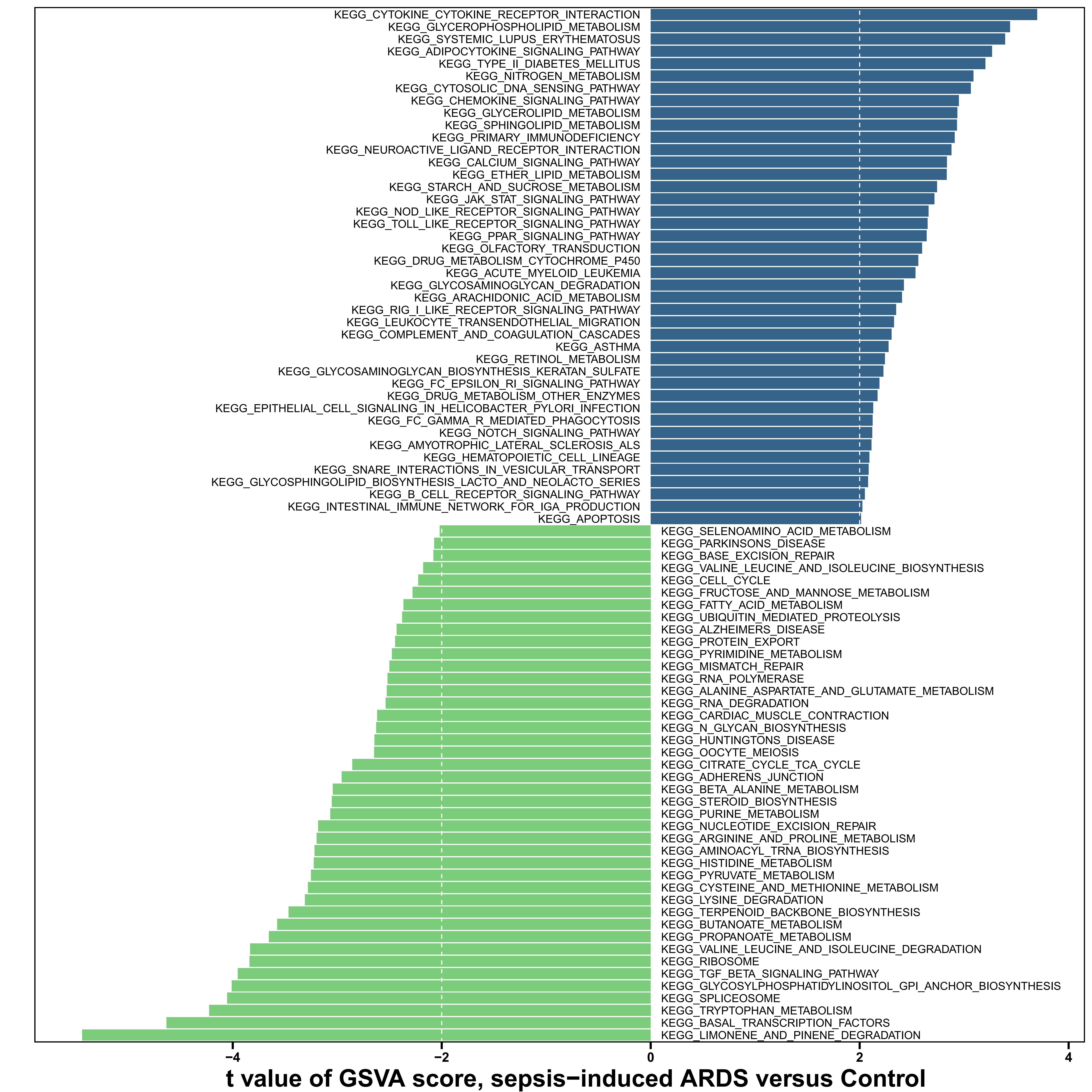
KEGG_CYTOKINE_CYTOKINE_RECEPTOR_INTERACTION
KEGG_GLYCEROPHOSPHOLIPID_METABOLISM
KEGG_SYSTEMIC_LUPUS_ERYTHEMATOSUS
KEGG_ADIPOCYTOKINE_SIGNALING_PATHWAY
KEGG_TYPE_II_DIABETES_MELLITUS
KEGG_NITROGEN_METABOLISM
KEGG_CYTOSOLIC_DNA_SENSING_PATHWAY
KEGG_CHEMOKINE_SIGNALING_PATHWAY
KEGG_GLYCEROLIPID_METABOLISM
KEGG_SPHINGOLIPID_METABOLISM
KEGG_PRIMARY_IMMUNODEFICIENCY
KEGG_NEUROACTIVE_LIGAND_RECEPTOR_INTERACTION
KEGG_CALCIUM_SIGNALING_PATHWAY
KEGG_ETHER_LIPID_METABOLISM
KEGG_STARCH_AND_SUCROSE_METABOLISM
KEGG_JAK_STAT_SIGNALING_PATHWAY
KEGG_NOD_LIKE_RECEPTOR_SIGNALING_PATHWAY
KEGG_TOLL_LIKE_RECEPTOR_SIGNALING_PATHWAY
KEGG_PPAR_SIGNALING_PATHWAY
KEGG_OLFACTORY_TRANSDUCTION
KEGG_DRUG_METABOLISM_CYTOCHROME_P450
KEGG_ACUTE_MYELOID_LEUKEMIA
KEGG_GLYCOSAMINOGLYCAN_DEGRADATION
KEGG_ARACHIDONIC_ACID_METABOLISM
KEGG_RIG_I_LIKE_RECEPTOR_SIGNALING_PATHWAY
KEGG_LEUKOCYTE_TRANSENDOTHELIAL_MIGRATION
KEGG_COMPLEMENT_AND_COAGULATION_CASCADES
KEGG_ASTHMA
KEGG_RETINOL_METABOLISM
KEGG_GLYCOSAMINOGLYCAN_BIOSYNTHESIS_KERATAN_SULFATE
KEGG_FC_EPSILON_RI_SIGNALING_PATHWAY
KEGG_DRUG_METABOLISM_OTHER_ENZYMES
KEGG_EPITHELIAL_CELL_SIGNALING_IN_HELICOBACTER_PYLORI_INFECTION
KEGG_FC_GAMMA_R_MEDIATED_PHAGOCYTOSIS
KEGG_NOTCH_SIGNALING_PATHWAY
KEGG_AMYOTROPHIC_LATERAL_SCLEROSIS_ALS
KEGG_HEMATOPOIETIC_CELL_LINEAGE
KEGG_SNARE_INTERACTIONS_IN_VESICULAR_TRANSPORT
KEGG_GLYCOSPHINGOLIPID_BIOSYNTHESIS_LACTO_AND_NEOLACTO_SERIES
KEGG_B_CELL_RECEPTOR_SIGNALING_PATHWAY
KEGG_INTESTINAL_IMMUNE_NETWORK_FOR_IGA_PRODUCTION
KEGG_APOPTOSIS
KEGG_SELENOAMINO_ACID_METABOLISM
KEGG_PARKINSONS_DISEASE
KEGG_BASE_EXCISION_REPAIR
KEGG_VALINE_LEUCINE_AND_ISOLEUCINE_BIOSYNTHESIS
KEGG_CELL_CYCLE
KEGG_FRUCTOSE_AND_MANNOSE_METABOLISM
KEGG_FATTY_ACID_METABOLISM
KEGG_UBIQUITIN_MEDIATED_PROTEOLYSIS
KEGG_ALZHEIMERS_DISEASE
KEGG_PROTEIN_EXPORT
KEGG_PYRIMIDINE_METABOLISM
KEGG_MISMATCH_REPAIR
KEGG_RNA_POLYMERASE
KEGG_ALANINE_ASPARTATE_AND_GLUTAMATE_METABOLISM
KEGG_RNA_DEGRADATION
KEGG_CARDIAC_MUSCLE_CONTRACTION
KEGG_N_GLYCAN_BIOSYNTHESIS
KEGG_HUNTINGTONS_DISEASE
KEGG_OOCYTE_MEIOSIS
KEGG_CITRATE_CYCLE_TCA_CYCLE
KEGG_ADHERENS_JUNCTION
KEGG_BETA_ALANINE_METABOLISM
KEGG_STEROID_BIOSYNTHESIS
KEGG_PURINE_METABOLISM
KEGG_NUCLEOTIDE_EXCISION_REPAIR
KEGG_ARGININE_AND_PROLINE_METABOLISM
KEGG_AMINOACYL_TRNA_BIOSYNTHESIS
KEGG_HISTIDINE_METABOLISM
KEGG_PYRUVATE_METABOLISM
KEGG_CYSTEINE_AND_METHIONINE_METABOLISM
KEGG_LYSINE_DEGRADATION
KEGG_TERPENOID_BACKBONE_BIOSYNTHESIS
KEGG_BUTANOATE_METABOLISM
KEGG_PROPANOATE_METABOLISM
KEGG_VALINE_LEUCINE_AND_ISOLEUCINE_DEGRADATION
KEGG_RIBOSOME
KEGG_TGF_BETA_SIGNALING_PATHWAY
KEGG_GLYCOSYLPHOSPHATIDYLINOSITOL_GPI_ANCHOR_BIOSYNTHESIS
KEGG_SPLICEOSOME
KEGG_TRYPTOPHAN_METABOLISM
KEGG_BASAL_TRANSCRIPTION_FACTORS
KEGG_LIMONENE_AND_PINENE_DEGRADATION
−4
−2
0
2
4
t value of GSVA score, sepsis−induced ARDS versus Control

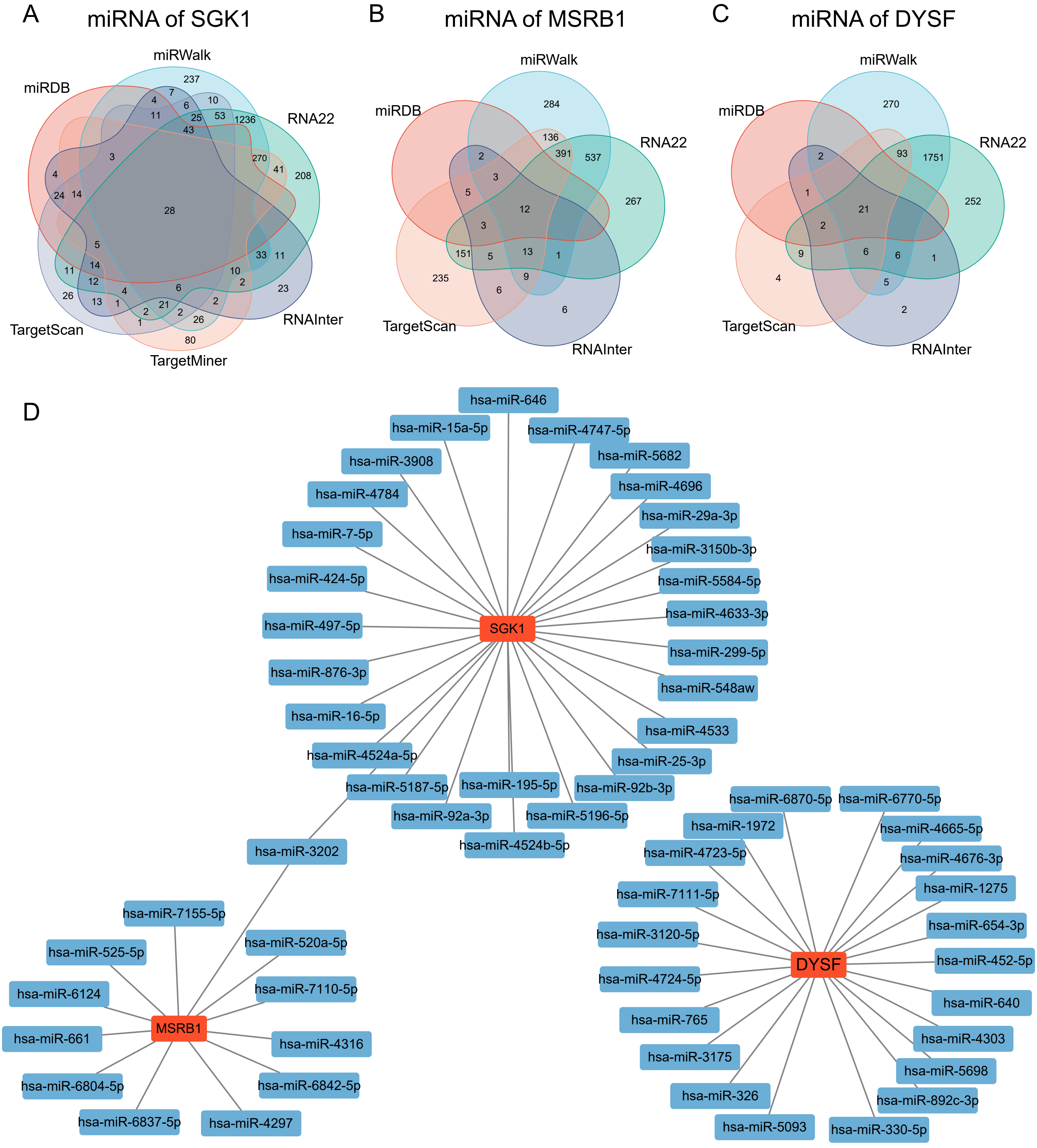
A
miRNA of SGK1
miRWalk
miRDB
RNA22
TargetScan
RNAInter
TargetMiner
B
miRNA of MSRB1
miRWalk
miRDB
RNA22
TargetScan
RNAInter
C
miRNA of DYSF
miRWalk
miRDB
RNA22
TargetScan
RNAInter
D
SGK1
hsa-miR-646
hsa-miR-15a-5p
hsa-miR-4747-5p
hsa-miR-3908
hsa-miR-5682
hsa-miR-4784
hsa-miR-4696
hsa-miR-29a-3p
hsa-miR-7-5p
hsa-miR-3150b-3p
hsa-miR-424-5p
hsa-miR-5584-5p
hsa-miR-4633-3p
hsa-miR-497-5p
hsa-miR-299-5p
hsa-miR-876-3p
hsa-miR-548aw
hsa-miR-16-5p
hsa-miR-4533
hsa-miR-4524a-5p
hsa-miR-25-3p
hsa-miR-5187-5p
hsa-miR-195-5p
hsa-miR-92b-3p
hsa-miR-92a-3p
hsa-miR-5196-5p
hsa-miR-4524b-5p
hsa-miR-3202
MSRB1
hsa-miR-7155-5p
hsa-miR-520a-5p
hsa-miR-525-5p
hsa-miR-7110-5p
hsa-miR-6124
hsa-miR-661
hsa-miR-4316
hsa-miR-6804-5p
hsa-miR-6842-5p
hsa-miR-6837-5p
hsa-miR-4297
DYSF
hsa-miR-6870-5p
hsa-miR-6770-5p
hsa-miR-1972
hsa-miR-4665-5p
hsa-miR-4723-5p
hsa-miR-4676-3p
hsa-miR-1275
hsa-miR-7111-5p
hsa-miR-654-3p
hsa-miR-3120-5p
hsa-miR-452-5p
hsa-miR-4724-5p
hsa-miR-640
hsa-miR-765
hsa-miR-4303
hsa-miR-3175
hsa-miR-5698
hsa-miR-326
hsa-miR-892c-3p
hsa-miR-5093
hsa-miR-330-5p
